# Efficacy and safety of unrestricted visiting policy for critically ill patients: a meta-analysis

**DOI:** 10.1186/s13054-022-04129-3

**Published:** 2022-09-05

**Authors:** Yuchen Wu, Guoqiang Wang, Zhigang Zhang, Luo Fan, Fangli Ma, Weigang Yue, Bin Li, Jinhui Tian

**Affiliations:** 1 Lanzhou University First Affiliated Hospital, Chengguan District, Lanzhou, 730000 Gansu Province China; 2 Lanzhou University Evidence Based Medicine Center, Lanzhou, 730000 China

**Keywords:** Delirium, ICU-acquired infection, Meta-analysis, Mortality, Restricted visiting policy, Unrestricted visiting policy

## Abstract

**Aim:**

To compare the safety and effects of unrestricted visiting policies (UVPs) and restricted visiting policies (RVPs) in intensive care units (ICUs) with respect to outcomes related to delirium, infection, and mortality.

**Methods:**

MEDLINE, Cochrane Library, Embase, Web of Science, CINAHL, CBMdisc, CNKI, Wanfang, and VIP database records generated from their inception to 22 January 2022 were searched. Randomized controlled trials and quasi-experimental studies were included. The main outcomes investigated were delirium, ICU-acquired infection, ICU mortality, and length of ICU stay. Two reviewers independently screened studies, extracted data, and assessed risks of bias. Random‑effects and fixed-effects meta‑analyses were conducted to obtain pooled estimates, due to heterogeneity. Meta-analyses were performed using RevMan 5.3 software. The results were analyzed using odds ratios (ORs), 95% confidence intervals (CIs), and standardized mean differences (SMDs).

**Results:**

Eleven studies including a total of 3741 patients that compared UVPs and RVPs in ICUs were included in the analyses. Random effects modeling indicated that UVPs were associated with a reduced incidence of delirium (OR = 0.4, 95% CI 0.25–0.63, *I*^2^ = 71%, *p* = 0.0005). Fixed-effects modeling indicated that UVPs did not increase the incidences of ICU-acquired infections, including ventilator-associated pneumonia (OR = 0.96, 95% CI 0.71–1.30, *I*^2^ = 0%, *p* = 0.49), catheter-associated urinary tract infection (OR 0.97, 95% CI 0.52–1.80, *I*^2^ = 0%, *p* = 0.55), and catheter-related blood stream infection (OR = 1.15, 95% CI 0.72–1.84, *I*^2^ = 0%, *p* = 0.66), or ICU mortality (OR = 1.03, 95% CI 0.83–1.28, *I*^2^ = 49%, *p* = 0.12). Forest plotting indicated that UVPs could reduce the lengths of ICU stays (SMD =  − 0.97, 95% CI − 1.61 to 0.32, *p* = 0.003).

**Conclusion:**

The current meta-analysis indicates that adopting a UVP may significantly reduce the incidence of delirium in ICU patients, without increasing the risks of ICU-acquired infection or mortality. Further large-scale, multicenter studies are needed to confirm these indications.

**Supplementary Information:**

The online version contains supplementary material available at 10.1186/s13054-022-04129-3.

## Introduction

The incidence of infections acquired in intensive care units (ICUs) is 2–5 times that in general wards [1, 2]. They complicate the regular hospitalization process, and are a major therapeutic issue that can compromise patients’ medical conditions (sometimes resulting in mortality), prolong treatment periods, and increase hospitalization costs [1, 3, 4]. Ventilator‑associated pneumonia (VAP), catheter-related blood stream infections (CRBSIs), and catheter-associated urinary tract infections (CAUTIs) are the most frequent ICU‑acquired infections. The estimated rate of mortality attributable to VAP is approximately 10%, with higher mortality rates in surgical ICU patients and patients with mid‑range severity scores at admission [4]. Restricted visiting policies (RVPs) may reduce the risk of infection in the vulnerable population of ICU patients [5–7]. Most ICUs have RVPs that define the number of visitors, visiting times, and other factors based on a unified hospital management policy and their ICU’s characteristics [8]. As more hospitals consider the benefits of family involvement in ICU holistic patient care, however, more ICUs are beginning to explore the advantages and disadvantages of adopting an unrestricted visiting policy (UVP) [9–11]. Growing evidence suggests that UVP implementation has positive effects on critical patient outcomes. Specifically, it can reduce the incidences of delirium, anxiety, and depression, and improve the satisfaction of patients and their families, without increasing the incidences of ICU-related infections or mortality [10–13]. Internationally restrictions on visitation in adult ICUs are common, with wide variability of reported policies [14].

Delirium is a substantial problem in critically ill patients, and it occurs in up to 83% of mechanically ventilated patients [15]. It is a well-recognized independent factor that is potentially detrimental to ICU patient outcomes [15–17]. It is associated with increases in mechanical ventilation time, prolonged ICU stays, and increased risks of falling and unplanned extubation. It is also associated with long-term cognitive impairment and increased mortality [16–18]. The types of visitations to ICUs can be categorized in several ways. RVPs do not meet the mental needs of patients and their families, and are a significant cause of patient suffering [10, 19, 20]. The risk of delirium in critically ill patients without family visits is increased by more than threefold, suggesting that family involvement can help prevent delirium in critically ill patients [21, 22]. Due to cultural variability and the unique treatment environment of ICUs, however, more than 70% of ICUs worldwide still implement RVPs with different visiting methods, times, frequencies, and numbers of visitors permitted [5, 8, 11]. The comparative effectiveness and safety of RVPs and UVPs remains uncertain. The aim of the current study was to compare the safety and effects associated with UVPs and RVPs in ICU patients with respect to outcomes related to mortality, infection, and delirium.

## Methods

The current study was conducted in accordance with the Preferred Reporting Items for Systematic Reviews and Meta-Analyses (PRISMA) statement and Cochrane Collaboration recommendations [23, 24], and the prespecified protocol was registered on PROSPERO (registration number CRD42020148782).

### Literature databases and search strategies

Two researchers (WG Y, YC W) searched the Cochrane Library, MEDLINE (PubMed), Embase, Cumulative Index to Nursing and Allied Health Literature (CINAHL), Web of Science, China Biology Medicine disk (CBMdisc), China National Knowledge Infrastructure (CNKI), Wanfang, and VIP databases in accordance with the PRISMA standards, from database inception until 22 January 2022, with no restrictions. The references lists of all retrieved publications were also checked in an effort to detect additional published studies.

Search terms included “visit,” “visiting,” “visitation,” “visitor,” “visitors,” “critical care,” “intensive care,” “burn units,” “NICU,” “MICU,” “EICU,” “SICU,” “RICU,” “recovery room,” “respiratory care unit,” and “ICU.” A combination of exploded Medical Subject Heading/Emtree terms along with “or” and “and” was used, as per the database specifications. The search strategy was developed by the author team and an evidence-based medicine expert (the search strategy in Additional file 1).

### Inclusion and exclusion criteria

#### Inclusion criteria

1. Randomized control trials (RCTs) or quasi-experimental studies (QEs) comparing the clinical effects of UVPs and RVPs.
2. Evaluated at least one of delirium, CRBSI, CAUTI, VAP, or mortality rate.
3. Used the Confusion Assessment Method for the ICU (CAM-ICU) scale to evaluate delirium [23].

#### Exclusion criteria

1. Review articles, case studies, or letters to editors
2. Pediatric studies
3. Duplicate references
4. Did not report relevant primary outcomes
5. Full text unavailable

### Data extraction

**Fig. 1:**
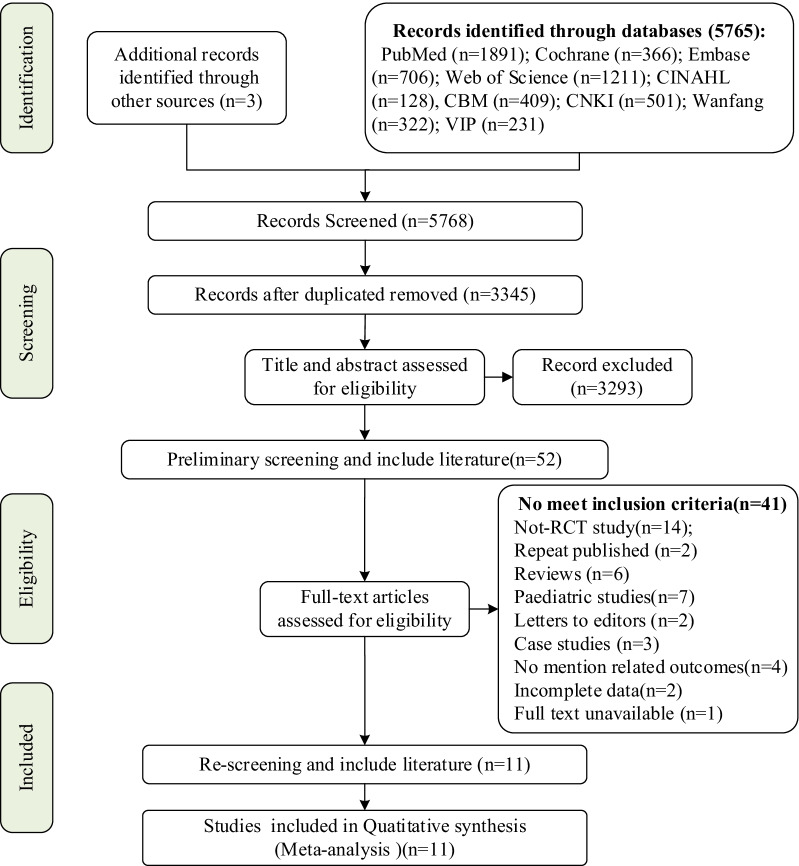
PRISMA flow diagram of the study selection process

Two researchers independently screened the titles and abstracts to evaluate the potential relevance of studies. Disagreements were resolved by consensus or discussion with a third author. After screening (Fig. 1), full-text reviews were performed. Detailed study information, interventions, controls, and outcomes were retrieved using a standardized data extraction protocol.

### Statistical analysis

Statistical analysis was conducted with Review Manager (RevMan) software (version 5.3, Cochrane Collaboration, Copenhagen, Denmark). For dichotomous variables (delirium, mortality, rate of ICU-acquired infection), odds ratios (ORs) and 95% confidence intervals (CIs) were calculated using the Mantel–Haenszel test. For continuous variables (length of ICU stay, anxiety, depression) the standardized mean difference (SMD) and 95% CI were calculated using the inverse-variance test. Chi-square test was applied to test heterogeneity (*p* < 0.05, *I*^2^ > 50%), and if it was found to exist, sensitivity analysis was applied to find out the cause. A random-effects model was used again if heterogeneity still could not be eliminated. Sensitivity analysis, subgroup analysis, or just descriptive analysis were applied when meeting significant clinical heterogeneity. A fixed-effects model was used for data with no significant heterogeneity (*p* ≥ 0.05, *I*^2^ ≤ 50%). The subgroup analysis will compare the study type included in all the included literature as sub-sites to see the source of heterogeneity. If ≥ 10 studies were present reporting bias was visually assessed via funnel plots. Pooled index significance was determined via the *Z* test. A two-sided *p* value of ≤ 0.05 was considered statistically significant.

### Assessment of risk of bias

A critical appraisal of each study was independently conducted by two reviewers using the JBI Critical Appraisal Checklists for Randomized Controlled Trials and Quasi-Experimental Studies [25]. Differences in opinion were resolved via discussion between the two reviewers, occasionally with arbitration by a third reviewer (JHT). To investigate study heterogeneity and test the robustness of results, sensitivity analyses omitting one study at a time were conducted. Two researchers used the Grading of Recommendations, Assessment, Development, and Evaluation (GRADE) approach for priority outcomes (GRADEpro version 3.6.1; GRADE Working Group 2004–2011) [26].

## Results

### Search outcomes

The search strategy identified 5768 relevant publications. After removing duplicate publications and screening the titles and abstracts, the remaining 3345 publications were rescreened. This process yielded 52 studies deemed appropriate for full review, of which 41 were subsequently excluded. Eleven studies [2, 11, 19, 27–34] were ultimately included in the current analysis (Fig. 1).

### Demographics of included studies

Eleven studies with a combined total of 3741 participants enrolled between 2006 and 2021 were included in the current investigation; 1868 assigned to a UVP group and 1873 assigned to an RVP group. Patient characteristics are shown in Table 1.

**Table 1:** Summary of the eligible studies

No.	Study	Country	Study design	Sample size		Mean age		Intervention		Outcomes
				UVP	RVP	UVP	RVP	UVP	RVP	
1	Xueping 2021	China	QE	82	82	63.8 ± 8.59	63.87 ± 7.43	The number and duration of visits were left to the patient’s preference, with the only restriction being a total time of 60 min	Single visitor per patient admitted for 20 min/d	①②⑤⑦
2	Xiliang 2020	China	QE	42	43	67.29 ± 7.19	64.58 ± 8.46	The number and duration of visits were left to the patient’s preference, with the only restriction being a total time of 60 min	Single visitor per patient admitted for 30 min/d	⑤⑦⑧⑨
3	Zhongxi 2020	China	QE	34	32	65.16 ± 12.6	65.7 ± 11.3	Two or fewer family visitors per patient at a time were allowed for up to 6 h/d, divided into six periods	Single visitor per patient admitted for 30 min/d	⑤⑥⑦⑧⑨
4	Rosa 2019	Brazil, SUA, Italy	RCT	837	848	58.4 ± 18.3	58.6 ± 18.2	Two or fewer visitors at a time were allowed for up to 12 h/d	Two or fewer visitors per patient at a time were allowed for up to 4.5 h/d, TID	①②③④⑤⑥⑦
5	Lifei 2018	China	RCT	177	178	54.93 ± 17.58	56.14 ± 16.79	The number and duration of visits were left to the patient’s preference	Single visitor per patient admitted for 30 min/d	①②③④⑤
6	Liping 2018	China	QE	85	71	60.08 ± 19.62	57.47 ± 18.53	The number and duration of visits were left to the patient’s preference, with the only restriction being a total time of 90 min	Two or fewer visitors per patient admitted for 30 min/d	⑤⑦
7	Xinying 2017	China	RCT	60	60	65 ± 3.6		The number and duration of visits were left to the patient’s preference	Single visitor per patient admitted for 20 min/d	⑤
8	Eghbali-Babadi M, 2017	Iran	RCT	34	34	55.11 ± 12.11	54.12 ± 13.11	The number and duration of visits were left to the patient’s preference	Single visitor per patient admitted for 30 min/d	⑤
9	Rosa 2017	Brazil	QE	145	141	60.5 ± 18.6	62.4 ± 20.6	Two or fewer visitors at a time were allowed for up to 12 h/d	Two or fewer visitors per patient at a time were allowed for up to 4.5 h/d, TID	①②③④⑤⑥⑦
10	Malacarne 2011	Italy	QE	261	269	60.7 ± 17.8	58.3 ± 21.1	Four visitors per patient were admitted for 90 min BID	Two visitors per patient admitted for 1 h/d	①②③④⑥⑦
11	Fumagalli 2006	Italy	RCT	111	115	68 ± 1	67 ± 1	The number and duration of visits were left to the patient’s preference, with the only restriction being one visitor at a time	Single visitor per patient admitted for 30 min BID	①②③④⑥⑧⑨

① ICU-acquired infection ② ventilator‑associated pneumonia ③ catheter-related blood stream infection ④ catheter-associated urinary tract infection ⑤ delirium ⑥ mortality ⑦ length of ICU stay ⑧ anxiety ⑨ depression

*RCT*—randomized control trial, *QE*—quasi-experimental, *UVP*—unrestricted visiting policy, *RVP*—restricted visiting policy, *BID* twice a day, *TID* three times a day

### Critical appraisal of included studies and GRADE assessment for priority outcomes

Five RCTs incorporated random allocation, without incomplete outcome data and without selective reporting or other biases [11, 19, 27, 28, 34]. Three RCTs incorporated blinding methods and allocation concealment [19, 27, 34]. One study was deemed to be of good overall quality [28], and three were deemed to be of fair overall quality [19, 27, 34]. The most frequent issue apart from blinding of participants and personnel was allocation concealment. Six QEs [2, 29–33] included a control group with similar participants and interventions, reliable measurements, appropriate statistical, clearly report “what is the cause and what is the effect” except follow-up that not applicable. Risks of bias assessment in RCTs are shown in Table 2, and risks of bias assessment in QEs are shown in Table 3. The numbers of RCTs reporting results for each priority outcome were low, and there was no indication of a small study effect that may have influenced the results. A GRADE assessment of the certainty of evidence is shown in Table 4.

**Table 2:** Critical appraisal of eligible randomized controlled trial study

Study	Year	Random allocation	Allocation concealment	Blind method	Incomplete outcome data	Selective reporting	Other bias
Fumagalli	2006	Low	Low	Low	Low	Low	Unclear
Xinying	2017	Low	Low	Low	Low	Low	Unclear
Eghbali-Babadi	2017	Low	Low	Low	Low	Low	Unclear
Lifei	2018	Low	Unclear	Unclear	Low	Low	Unclear
Rosa	2019	Low	Unclear	High	Low	Low	Unclear

**Table 3:** Critical appraisal of eligible quasi-experimental studies

Study	Year	Is it clear in the study what is the “cause” and what is the “effect”	Were the participants included in any comparisons similar?	Were the participants included in any comparisons receiving similar intervention	Was there a control group?	Were there multiple measurements of the outcome both pre and post the intervention?	Was follow-up complete, and if not were differences between groups in terms of their follow-up adequately described and analyzed?	Were the outcomes of participants included in any comparisons measured in the same way?	Were outcomes measured in a reliable way	Was appropriate statistical analysis used
Rosa	2017	Yes	Yes	Yes	Yes	Yes	Not applicable	Yes	Yes	Yes
Liping	2018	Yes	Yes	Yes	Yes	Yes	Not applicable	Yes	Yes	Yes
Malacarne	2011	Yes	Yes	Yes	Yes	Yes	Not applicable	Yes	Yes	Yes
Xueping	2021	Yes	Yes	Yes	Yes	Yes	Not applicable	Yes	Yes	Yes
Xiliang	2020	Yes	Yes	Yes	Yes	Yes	Not applicable	Yes	Yes	Yes
Zhongxi	2020	Yes	Yes	Yes	Yes	Yes	Not applicable	Yes	Yes	Yes

**Table 4:** Summary of findings and GRADE assessment for priority outcomes

**UVP compared to RVP for critical patients**				
**Patient or population:** critically ill patients				
**Settings:** Intensive care unit				
**Intervention:** UVP				
**Comparison:** RVP				
Outcomes	No. of participants (significant studies)	Relative effect (95% CI)	Quality of the evidence (GRADE)	Comments
ICU-acquired infection	3246 (3RCTs; 3 QEs)	OR 0.92 (0.72 to 1.18)	⊕⊕⊝⊝ Low^a,b,c^	Downgraded two levels due to inconsistency and imprecision
VAP	3246 (3RCTs; 3 QEs)	OR 0.96 (0.71 to 1.3)	⊕⊕⊝⊝ Low^a,b,c^	Downgraded two levels due to inconsistency and imprecision
CAUTI	3082 (3RCTs; 2 QEs)	OR 0.97 (0.52 to 1.8)	⊕⊕⊝⊝ Low^a,b,c^	Downgraded two levels due to inconsistency and imprecision
CRBSI	3082 (3RCTs; 2 QEs)	OR 1.15 (0.72 to 1.84)	⊕⊕⊝⊝ Low^a,b,c^	Downgraded two levels due to inconsistency and imprecision
Delirium	2985 (4RCTs; 5 QEs)	OR 0.4 (0.25 to 0.63)	⊕⊕⊕⊝ Moderate^a,b,c^	Downgraded one level due to inconsistency
Mortality	2727 (2RCTs; 2 QEs)	OR 1.03 (0.83 to 1.28)	⊕⊕⊕⊝ Moderate^a,b,c^	Downgraded one levels due to inconsistency
ICU length of stay	2972 (1RCTs; 6 QEs)	SMD − 0.81 (− 1.3 to − 0.32)	⊕⊕⊝⊝ Low^c^	Downgraded two levels due to risk of bias, and indirectness
Anxiety	311 (1RCTs; 1 QEs)	SMD − 2.39 (− 5.03 to 0.25)	⊕⊕⊝⊝ Low^a,b^	Downgraded two levels due to inconsistency and imprecision
Depression	311 (1RCTs; 1 QEs)	SMD − 2.1 (− 3.22 to − 0.97)	⊕⊕⊝⊝ Low^a,b^	Downgraded two levels due to inconsistency and imprecision

*CI*—confidence interval, *OR*—odds ratio, *RCTs*—randomized controlled trial study, *QEs*—quasi-experiment study

^a^Inconsistently visiting hours

^b^Inconsistent number of visitors

^c^Big sample size in one study; sufficient sample size in the others

### Delirium

**Fig. 2:**
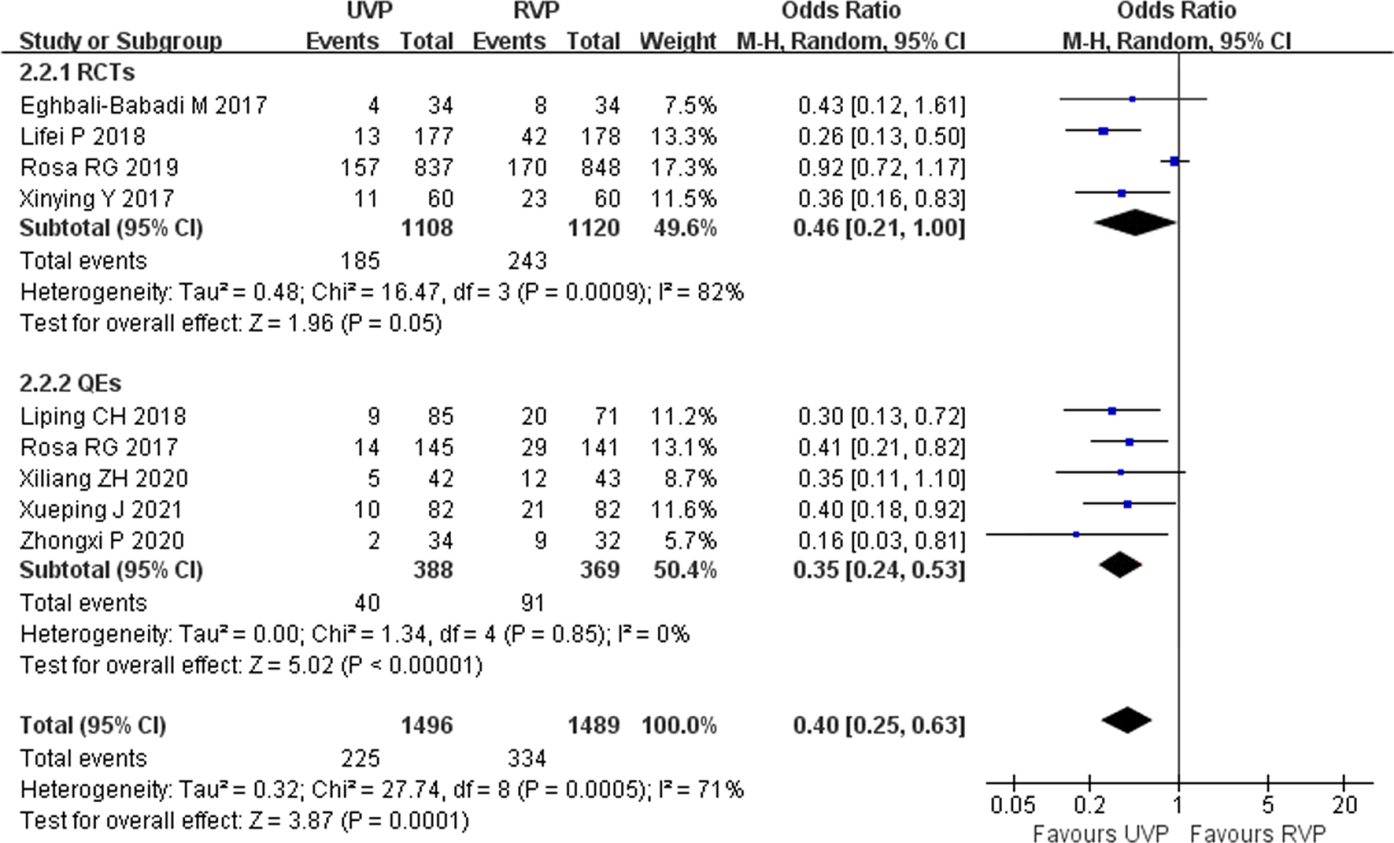
Forest plot of eligible studies that reported delirium

Nine studies including a total of 2975 patients investigated the effects of UVPs on the incidence of delirium, including four RCTs [11, 19, 27, 28] and five QEs [2, 29–32]. Subgroup analysis was performed using the type of study. According to the heterogeneity test results (*p* = 0.0005, *I*^2^ = 71%) the level of heterogeneity was high. Subgroup analysis was conducted according to research method, and a random effects model was used for the meta-analysis, and the overall meta-analysis showed that UVPs could reduce the incidence of delirium (OR = 0.40, 95% CI 0.25–0.63, *p* = 0.0001) (Fig. 2). The heterogeneity of each study was low.

### ICU-acquired infection

**Fig. 3:**
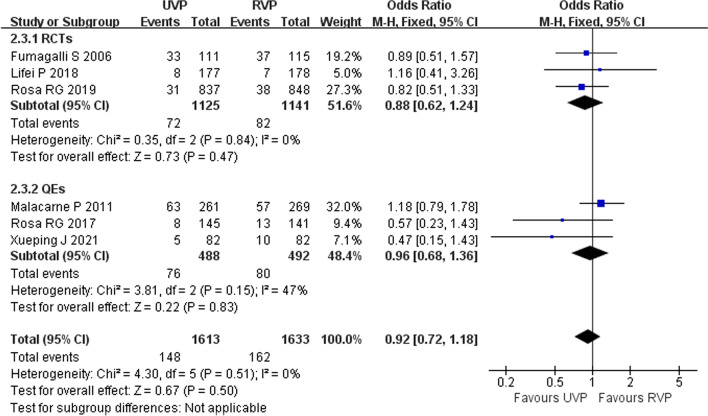
Forest plot of eligible studies that reported ICU-acquired infection

**Fig. 4:**
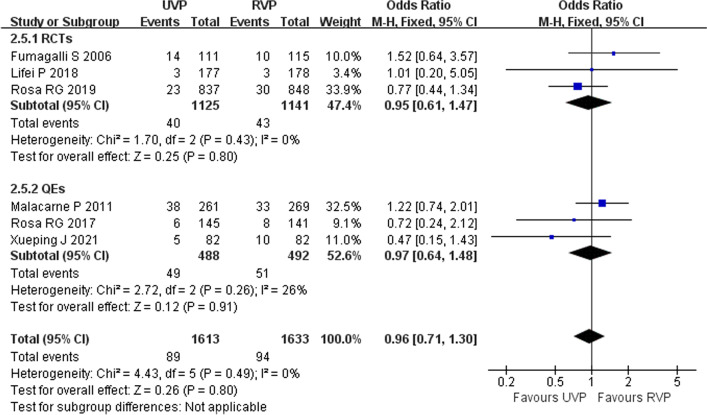
Forest plot of eligible studies that reported VAP

Six studies including a total of 3246 patients investigated relationships between UVPs and infections acquired in ICU patients, three RCTs [11, 28, 34] and three QEs [2, 29, 33]. Study type subgroup analysis was performed. Overall meta-analysis was performed using a fixed-effects model because the heterogeneity of each study was low (*p* = 0.51, *I*^2^ = 0%). UVP had no influence on ICU-acquired infection of patients (OR = 0.92, 95% CI 0.72–1.18, *p* = 0.5) (Fig. 3). Six studies investigated the effects of UVPs on the incidence of VAP. Study type subgroup analysis was performed. Overall meta-analysis using a fixed-effects model showed that UVPs did not increase the incidence of VAP (*I*^2^ = 0%, *p* = 0.49, OR = 0.96, 95% CI 0.71–1.30, *p* = 0.80) (Fig. 4).

**Fig. 5:**
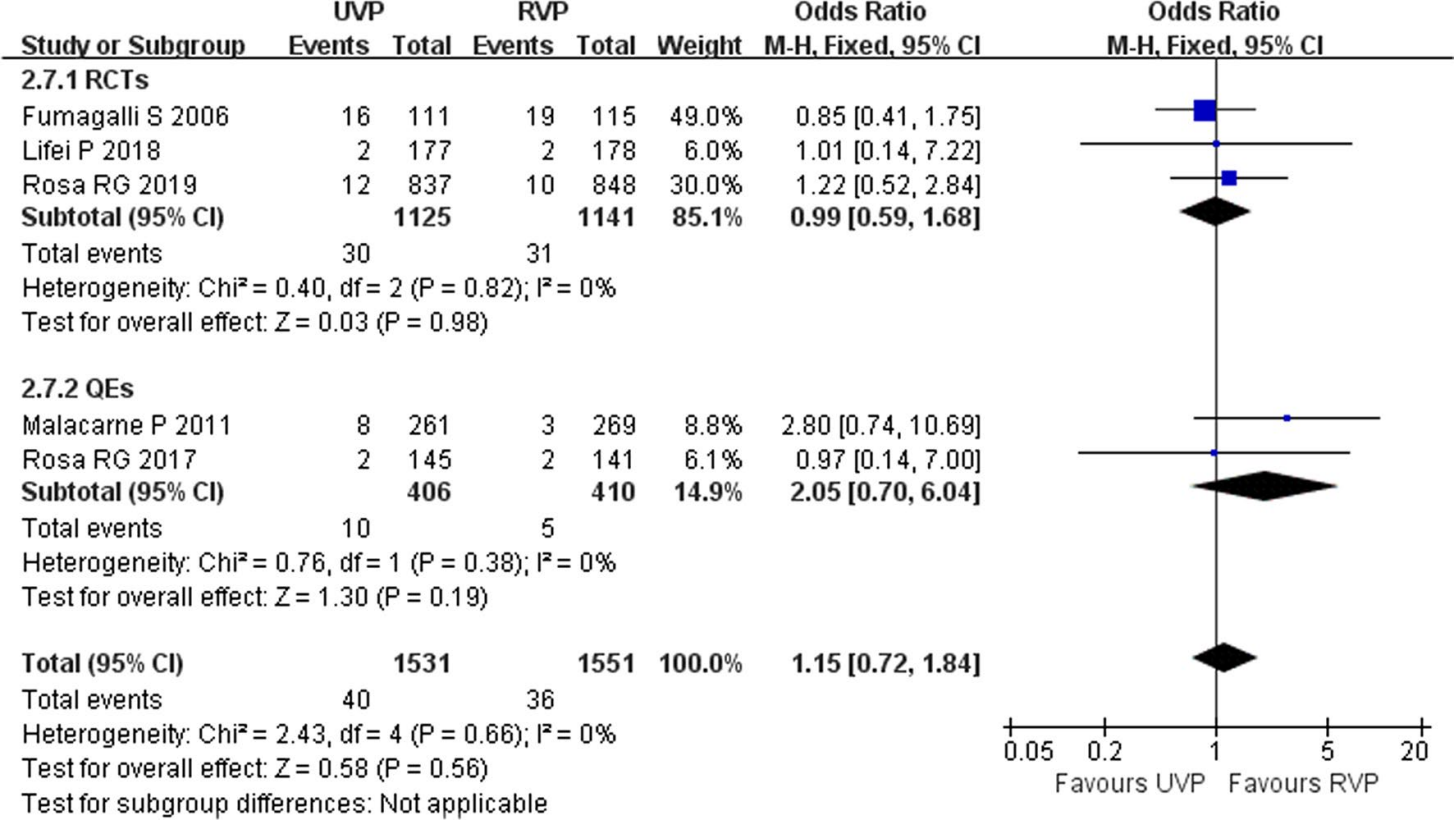
Forest plot of eligible studies that reported CRBSI

**Fig. 6:**
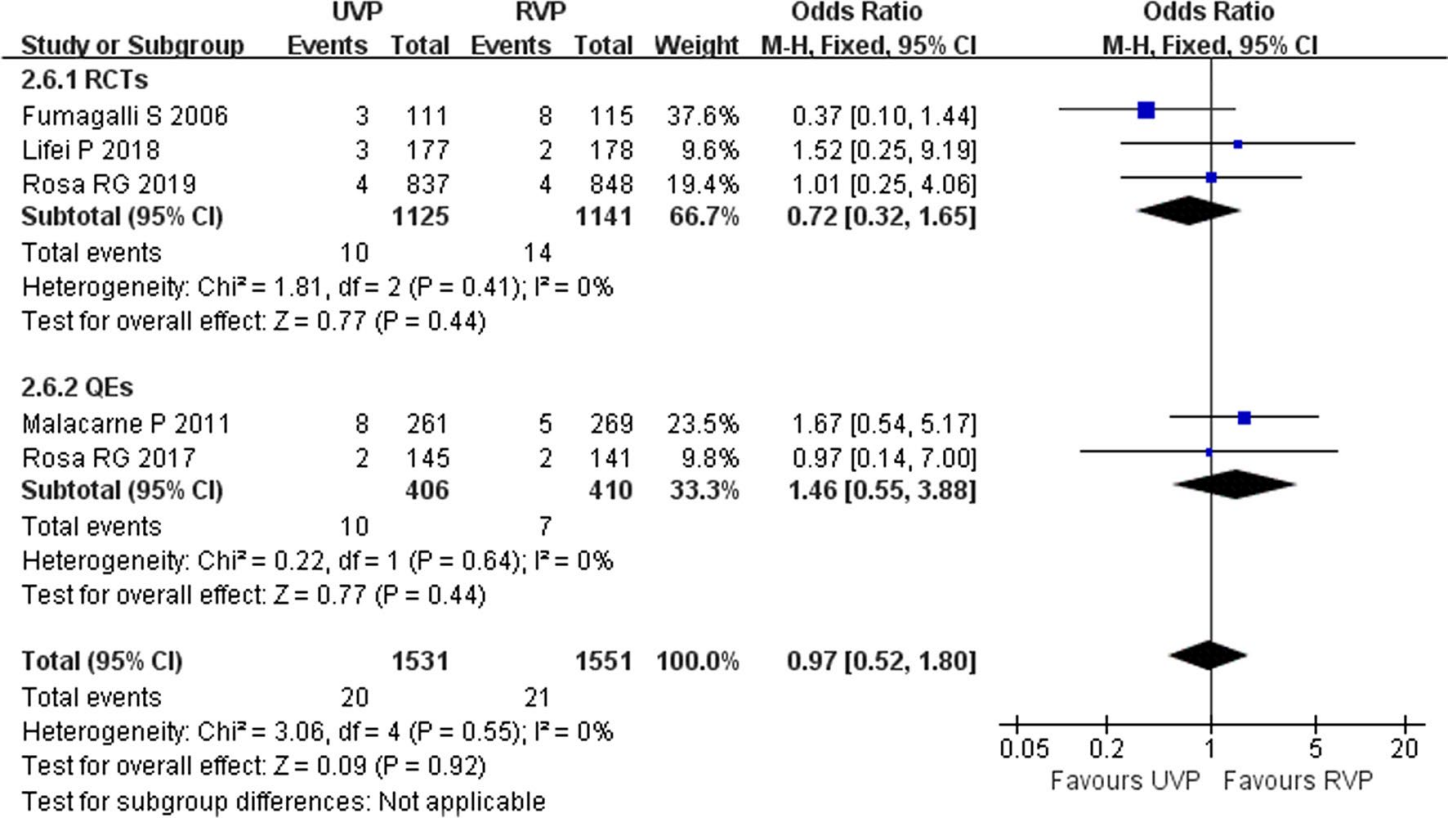
Forest plot of eligible studies that reported CAUTI

Five studies (three RCTs [11, 28, 34] and two QEs [2, 33]) including 3082 patients reported CRBSIs and CAUTIs in ICU patients. Heterogeneity test results of CRBSI (*p* = 0.66, *I*^2^ = 0%) and CAUTI (*p* = 0.55, *I*^2^ = 0%) were acceptable, and a fixed-effects model was used in the meta-analysis. UVPs did not increase the incidence of CRBSI (OR 1.15, 95% CI 0.72–1.84, *p* = 0.56) (Fig. 5) or CAUTI (OR 0.97, 95% CI 0.52–1.80, *p* = 0.92) (Fig. 6).

### ICU mortality rate

**Fig. 7:**
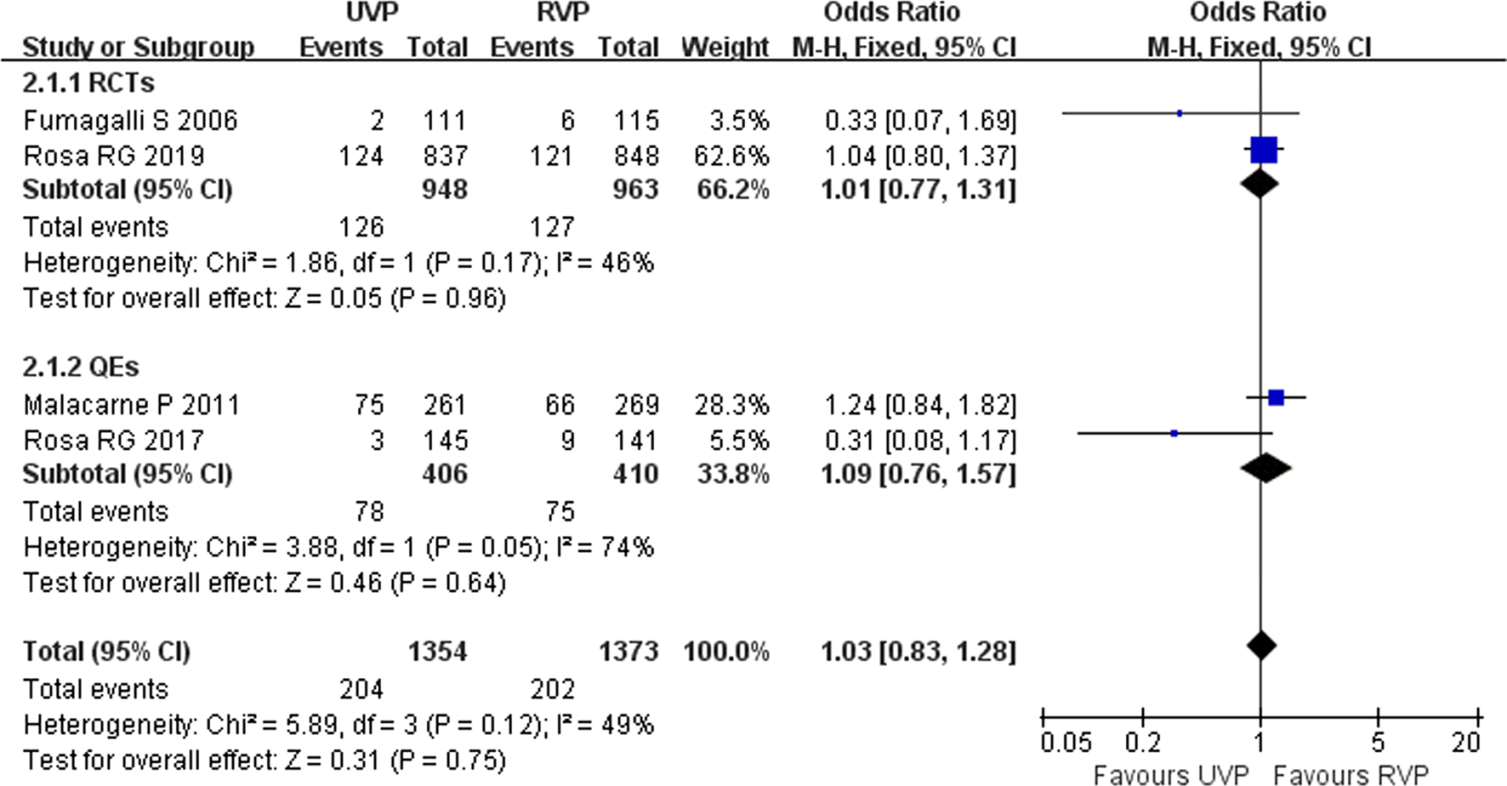
Forest plot of the eligible studies that reported mortality

Four studies (two RCTs [11, 34] and two QEs [2, 33]) investigated the effects of UVPs on the ICU mortality rate in 2727 patients, and UVPs did not significantly increase the ICU mortality rate (*I*^2^ = 49%, *p* = 0.12, fixed effect model; OR = 1.03, 95% CI 0.83–1.28, *p* = 0.75) (Fig. 7).

### ICU length of stay

**Fig. 8:**
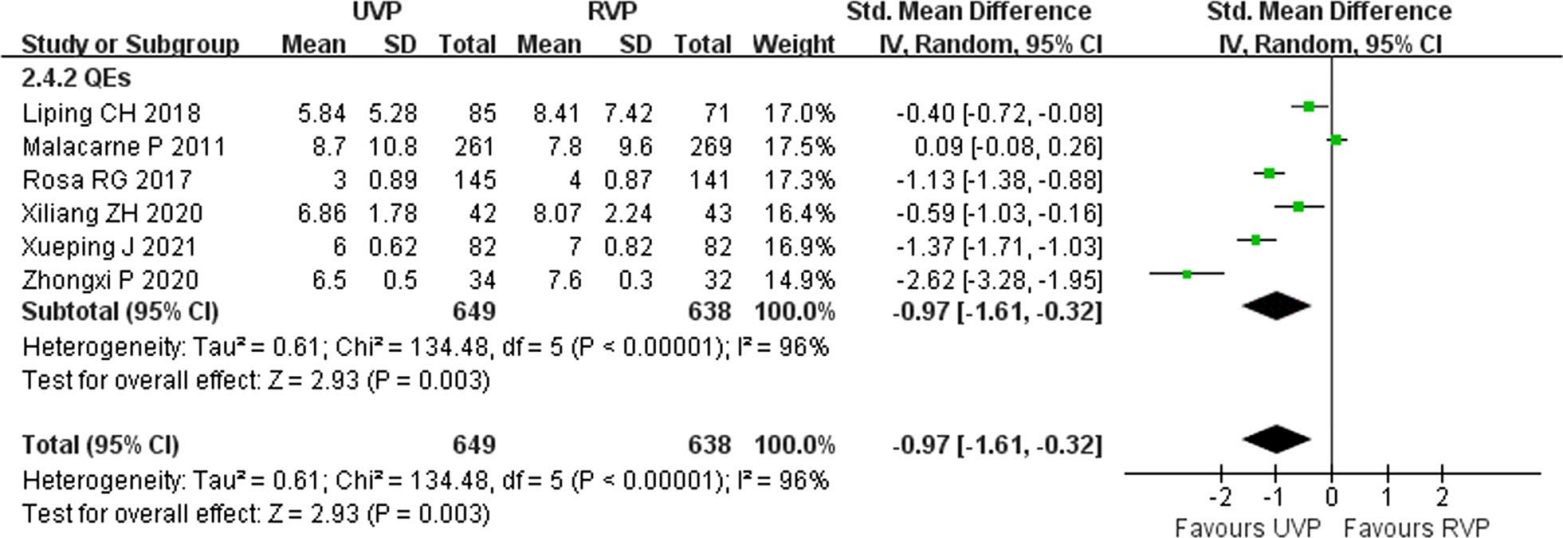
Forest plot of eligible studies that reported lengths of ICU stays

Seven studies (one RCT [11] and six QEs [2, 29–33]) including 2972 patients reported ICU length of stay (LoS). The level of heterogeneity was high (*p* < 0.00001, *I*^2^ = 97%), and the random effects model was used in the meta-analysis. Pooled analysis of the data indicated that UVPs could reduce the lengths ICU of stays (SMD =  − 0.97, 95% CI − 1.61 to − 0.32, *p* = 0.003) (Fig. 8).

### Anxiety and depression

**Fig. 9:**
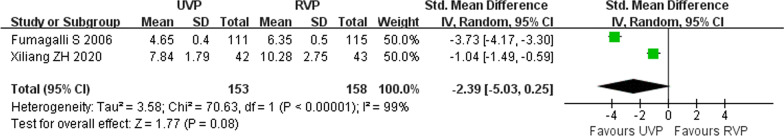
Forest plot of eligible studies that reported anxiety

**Fig. 10:**
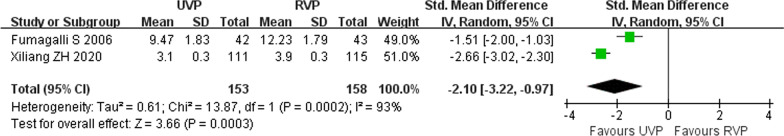
Forest plot of eligible studies that reported depression

Two studies used the Hamilton Anxiety Scale and the Hamilton Depression Scale to evaluate patient anxiety and depression [30, 34], and one study used the Self-Rating Anxiety Scale and the Self-Rating Depression Scale to evaluate patient anxiety and depression [31]. Due to large differences in mean anxiety and depression the SMD and the effect size were calculated, and the heterogeneity test results were *p* < 0.00001, *I*^2^ = 99% for anxiety and *p* = 0.0002, *I*^2^ = 93% for depression. A random effects model was used for the meta-analysis. Patient anxiety scores were better in the UVP group than in the control group, but not statistically significantly (SMD =  − 2.39, 95% CI − 5.03 to 0.25, *p* = 0.08) (Fig. 9). There was a significant reduction in Hamilton Depression Scale scores associated with UVPs in the random effects model (SMD − 2.1, 95% CI − 3.22 to − 0.97, *p* = 0.0003, *I*^2^ 93%) (Fig. 10).

### Funnel plot

**Fig. 11:**
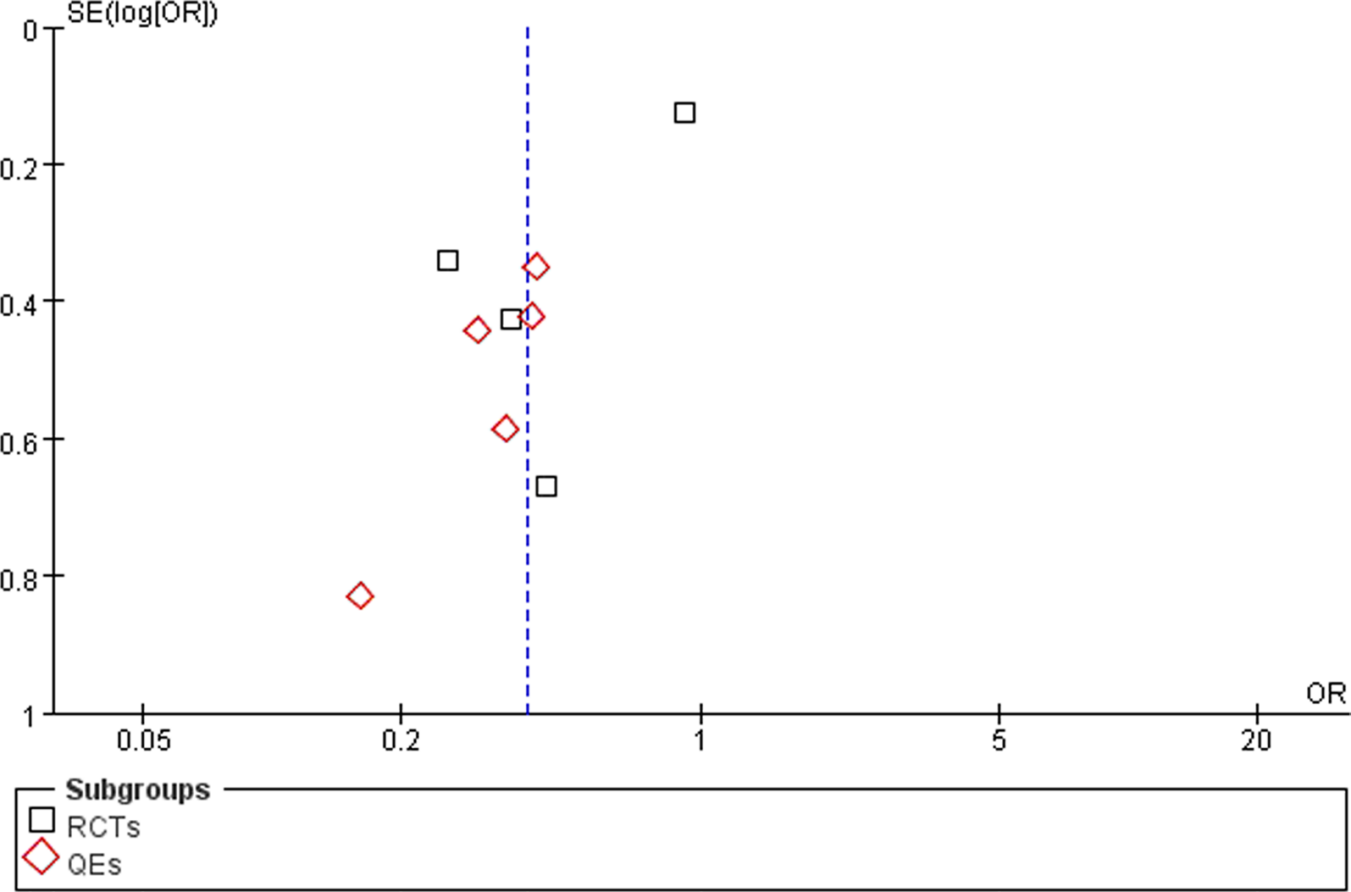
Funnel plot of the primary outcome of delirium

Funnel plots were drawn with the primary outcome of delirium, and the results showed risk bias. We further conducted sensitivity analyses to explore heterogeneity, which related with a big sample research [11] (Fig. 11). We planned to conduct heterogeneity assessment based on predefined factors (study type, sample size) and to assess small study effects using funnel plots and Egger’s test, where appropriate. However, the number of studies for each reported outcome was too low to allow a meaningful assessment.

## Discussion

“Being close” is one of the most basic and important needs of family members of critically ill patients. UVPs provide an increased opportunity to be at the bedside with the patient, but they are not universally embraced by adult intensive care units worldwide [13]. The current meta-analysis suggests that compared with RVPs, UVPs are associated with a lower incidence of delirium and no increased risk of ICU mortality or ICU-acquired infection. This conclusion is consistent with the results of Nassar et al. [10] and also supports the concept of an “open” ICU in which family visits are unrestricted based on “humanistic care” and a “patient-centered” approach, in line with the latest guidelines, comments, expert consensus, and conference reports [2, 8].

It has been authoritatively stated that delirium is strongly associated with mortality [16–18], and mortality was almost the same in the RVP and UVP groups, which is associated with the following factors. Firstly, only four studies reported mortality but nine studies reported delirium. Secondly, the duration of visits, number of visitors during each visit, and visitation frequency varied across studies with respect to times of the day. Thirdly, because the heterogeneity of delirium-related studies was high (*I*^2^ = 71%, *p* = 0.0005) there was a significant difference in the incidence of delirium in RVP and UVP groups (OR = 0.40, 95% CI 0.25–0.63, *p* = 0.0001). This may be related to the large sample size of the study by Rosa et al. [11], and inclusion of QEs which affected the effect sizes.

More than half of critically ill patients experience anxiety and depression symptoms [35], and the incidence of delirium in response to sudden mental symptoms in this population ranges from 38.9 to 77.4% [36–38]. The incidence of delirium in ICU patients with restricted visitation was reported to be more than threefold that of patients with unrestricted visitation [39]. A UVP is a humanized service aimed at reducing separation anxiety caused by a closed ICU. It can provide comfort and a sense of security to critically ill patients [12, 34, 40]. Studies have found that UVPs can help patients establish contact with the outside world, giving them courage and confidence to fight against their condition [5, 12]. Secondly, visitors can provide mental and social support and relieve patients’ negative emotions during treatment, all of which protect against stress [10, 41]. Lastly, family involvement can reduce the need for analgesics and sedatives, reduce the incidence of ICU syndrome, and shorten ICU stays [19, 42, 43]. Collectively, our findings suggest that UVPs should be implemented in ICUs so that family members can participate in the psychological, social, and emotional support of critically ill patients in a timely manner to promote their reorientation, cognitive health, and rehabilitation.

Critical patients are frail and prone to cross-infection, and one of the most frequent objections to UVPs in ICUs is the risk of an increased rate of acquired infections. Although there is no evidence of an increased incidence of infections in open ICUs, several surveys have expressed caregivers’ concerns that visitors could bring infections into the units through a form of ‘‘pollination’’ [33]. Therefore, visitors are required to practice good hand hygiene and wear disposable isolation clothes and/or personal protective equipment when appropriate [2, 33]. Hand hygiene is an economical, simple, unique, and effective measure for controlling nosocomial infection [3, 7, 44]. A World Health Organization review found that baseline compliance with hand hygiene among healthcare workers was on average only 38.7% (range 5–89%) [44]. Multicomponent interventions are effective in improving hand hygiene compliance, and improved hand hygiene compliance can reduce the rate of hospital-acquired infections and catheter-associated urinary tract infections [4, 45]. However, due to busy schedules, improper hand washing, insufficient equipment, and other reasons, hand hygiene implementation by medical staff still needs improvement [3]. Similarly, when we asked family members why they did not wash their hands in accordance with hospital procedures one replied that “the total visit time is 20 min, hand hygiene takes too much time, and our other family members also want to visit.” With RVPs, family members want to provide more emotional support to patients in a short period and therefore ignore hand hygiene. In contrast, UVPs provide time, so family members may be more likely to perform the hand washing protocol. This may be why UVPs do not increase the incidence of ICU-acquired infections.

More liberal visiting policies seem to be safe for patients with regard to the risks of ICU mortality and LoS. There was no increase in the LoS in the combined analysis of data from the four studies that reported on this outcome [2, 29–33]. Although no difference was observed in ICU mortality, in the largest included study, units with lower standardized mortality ratios were also those with more liberal visiting policies [13].

Visiting hours vary among countries, due to national, cultural, and religious differences. This confounding factor could not be addressed in the meta-analysis. Compared with developing countries, ICU visitation policies in developed countries are more flexible and allow relatively long visits [2, 5, 10, 46–48]. The median number of visiting hours was > 4/d in ICUs in Brazil, USA, and Italy [11, 12, 49], and visiting hours ranged from 1.5 to 24.0 h/d in the Netherlands [14], compared to just 1–2 h/d in Iran [19]. The results of this meta-analysis should be interpreted with caution. Firstly, despite a comprehensive database search, only a five RCTs were eligible for inclusion. Our study demonstrated that there is high heterogeneity in visitation policies among ICUs in different countries. But, it is unable to address potential effect modifiers at country level. Secondly, the concept of UVP varies between studies and countries, which may related to different periods of cultural and organizational aspects. Thirdly, our study was only possible for some patient-related outcomes, and most of the results showed high heterogeneity. Consequently, we need to implement UVP with critical thinking.

## Conclusion

The current meta-analysis compared the efficacy and safety of UVPs and RVPs for adult ICU patients, and the conclusions were based on moderate-certainty evidence. The results indicate that UVPs can reduce the incidence of delirium in ICU patients, shorten the lengths of ICU stays, and reduce anxiety and depression scores, without increasing rates of ICU-acquired infection. Based on this, we suggest that ICUs should implement UVPs.

## Supplementary Information

**Additional file 1.** Search strategy.

## Data Availability

Data can be requested from the Ethics Committee of the First Hospital of Lanzhou University, Lanzhou, Gansu, China (email ldyylwh@126.com) by researchers who meet the criteria for access to confidential data.
