# Supplementary material for: Efficacy and safety of unrestricted visiting policy for critically ill patients: a meta-analysis
Source: Crit Care. 2022 Sep 5;26:267. doi: 10.1186/s13054-022-04129-3 (PMC9446669; doi:10.1186/s13054-022-04129-3)
Supplement: Supplementary file 1 — Additional file 1. Search strategy. [file 13054_2022_4129_MOESM1_ESM.docx]

| Pubmed: Search: **#1 and #4** |
| --- |
| ("intensive care unit"[Title/Abstract] OR "intensive care units"[Title/Abstract] OR "burn units"[Title/Abstract] OR "burn unit"[Title/Abstract] OR "coronary care units"[Title/Abstract] OR "coronary care unit"[Title/Abstract] OR "ICU"[Title/Abstract] OR "recovery room"[Title/Abstract] OR "respiratory care units"[Title/Abstract] OR "respiratory care unit"[Title/Abstract] OR "SICU"[Title/Abstract] OR "EICU"[Title/Abstract] OR "RICU"[Title/Abstract] OR "MICU"[Title/Abstract] OR "NICU"[Title/Abstract]) AND ("visitation"[Title/Abstract] OR "visiting"[Title/Abstract] OR "visit"[Title/Abstract] OR "Familys"[Title/Abstract] OR "visitor"[Title/Abstract] OR "visitors"[Title/Abstract]) |

| **Embase: Search: #1 and #2** |
| --- |
| ('intensive care unit':ti,ab,kw OR 'intensive care units':ti,ab,kw OR 'burn units':ti,ab,kw OR 'burn unit':ti,ab,kw OR 'coronary care units':ti,ab,kw OR 'coronary care unit':ti,ab,kw OR 'ICU':ti,ab,kw OR 'recovery room':ti,ab,kw OR 'respiratory care units':ti,ab,kw OR 'respiratory care unit':ti,ab,kw OR 'SICU':ti,ab,kw OR 'EICU':ti,ab,kw OR 'RICU':ti,ab,kw OR 'MICU':ti,ab,kw OR 'NICU':ti,ab,kw) AND ('visitation':ti,ab,kw OR 'visiting':ti,ab,kw OR 'visit':ti,ab,kw OR 'Familys':ti,ab,kw OR 'visitor':ti,ab,kw OR 'visitors':ti,ab,kw) |

| **Cochrane Library: #1 AND #2** |
| --- |
| ((intensive care unit):ti,ab,kw OR (intensive care units):ti,ab,kw OR (burn units):ti,ab,kw OR (burn unit):ti,ab,kw OR (coronary care units):ti,ab,kw OR (coronary care unit):ti,ab,kw OR (ICU):ti,ab,kw OR (recovery room):ti,ab,kw OR (respiratory care units):ti,ab,kw OR (respiratory care unit):ti,ab,kw OR (SICU):ti,ab,kw OR (EICU):ti,ab,kw OR (RICU):ti,ab,kw OR (MICU):ti,ab,kw OR (NICU):ti,ab,kw) AND ((visitation):ti,ab,kw OR (visiting):ti,ab,kw OR (visit):ti,ab,kw OR (Familys):ti,ab,kw OR (visitor):ti,ab,kw OR (visitors):ti,ab,kw) |
